# Hospital point-of-use water filtration to prevent exposure to waterborne pathogens

**DOI:** 10.1186/1753-6561-5-S6-P310

**Published:** 2011-06-29

**Authors:** SM Camps, AJ Rijs, B de Graaf, AH Paulitsch, PE Verweij, A Voss

**Affiliations:** 1 Medical Microbiology, Radboud University Nijmegen Medical Centre, Nijmegen, Netherlands; 2 Vitens Laboratories, Netherlands; 3 Wetsus, Leeuwarden, Netherlands

## Introduction / objectives

Hospital water systems have regularly been shown to serve as a reservoir for waterborne pathogens such as *Legionella* spp, *Mycobacteria* spp and fungi, all able to cause life-threatening infections especially in immunocompromised patients. In this study, the suitability of new point-of-use water filters was evaluated in a clinical setting.

## Methods

During a routine control of hospital water, contamination with *Legionella* spp*.* was detected. To protect the patients from exposure to *Legionella*, shower heads were replaced by point-of-use shower filters (H2OK medical filters, Norit Filtrix, The Netherlands). The efficacy of 4 shower filters was tested during 5 weeks. Water samples were taken with and without use of the shower filter and were analyzed for the presence of *Legionella*, heterotrophic plate count (HPC), and fungi. After 5 weeks of use, shower filters were examined using scanning electron microscopy (SEM).

## Results

96% of the samples taken without the filter were positive for *Legionella* whereas all filtered water samples were *Legionella* free. Although other bacteria were present in the filtered water, the HPC was significantly reduced compared to the unfiltered water. *Fusarium oxysporum* and *Fusarium solani* were regularly detected in samples taken without shower filter but were never observed in filtered samples. SEM analysis showed a variety of structures on the inner side of the filter membranes while the outside of the membrane did not show any changes compared to an unused control filter.

## Conclusion

The point-of-use filters proved to be highly effective in eliminating potentially pathogenic *Legionella* and *Fusarium* species from the showering water. The effects were seen over the complete 5 week study period.

## Disclosure of interest

None declared.

